# Fibroblast growth factor 19 entry into brain

**DOI:** 10.1186/2045-8118-10-32

**Published:** 2013-11-01

**Authors:** Hung Hsuchou, Weihong Pan, Abba J Kastin

**Affiliations:** 1 Blood-Brain Barrier Group, Pennington Biomedical Research Center, 6400 Perkins Road, Baton Rouge, LA 70808, USA

**Keywords:** FGF19, FGF15, Blood–brain barrier, Liver, Kidney

## Abstract

**Background:**

Fibroblast growth factor (FGF)-19, an endocrine FGF protein mainly produced by the ileum, stimulates metabolic activity and alleviates obesity. FGF19 modulates metabolism after either intravenous or intracerebroventricular injection, and its receptor FGFR4 is present in the hypothalamus. This led to the question whether blood-borne FGF19 crosses the blood-brain barrier (BBB) to exert its metabolic effects.

**Methods:**

We determined the pharmacokinetics of FGF19 permeation from blood to brain in comparison with its distribution in peripheral organs. Multiple-time regression analysis after intravenous bolus injection, in-situ brain perfusion, and HPLC assays were performed.

**Results:**

FGF19 was relatively stable in blood and in the brain compartment. Significant influx was seen in the presence of excess unlabeled FGF19 in blood. This coincided with a slower decline of ^125^I-FGF19 in blood which suggested there was decreased clearance or peripheral tissue uptake. In support of an altered pattern of peripheral processing of ^125^I-FGF19 by excess unlabeled FGF19, the high influx to liver was significantly attenuated, whereas the minimal renal uptake was linearly accelerated. In the present setting, we did not detect a saturable transport of FGF19 across the BBB, as the entry rate of ^125^I-FGF19 was not altered by excess unlabeled FGF19 or its mouse homologue FGF15 during in-situ brain perfusion.

**Conclusion:**

FGF19 remained stable in the blood and brain compartments for up to 10 min. Its influx to the brain was non-linear, non-saturable, and affected by its blood concentration and distribution in peripheral organs. Liver showed a robust and specific uptake of FGF19 that could be inhibited by the presence of excess unlabeled FGF19, whereas kidney clearance was dose-dependent.

## Introduction

Mouse fibroblast growth factor (FGF)-15 and its human ortholog FGF19 are endocrine hormones mainly produced by the ileum to inhibit bile acid synthesis and regulate the metabolism of lipids and glucose [1,2]. A beneficial role of these polypeptides in metabolic signaling is suggested by the higher energy expenditure, reduced adiposity, and resistance to diet-induced obesity of FGF19 transgenic mice [3]. Direct treatment of animals with FGF19 by either intravenous or intracerebroventricular injection also produces similar effects of increased energy expenditure [4]. Brown adipose tissue and liver are major mediators of FGF19 in the periphery. FGF15 is expressed in the developing brain but appears to be absent in adults [5]. It has been suggested that FGF19 acts on the brain. FGF19 reduces 24 h food intake, body weight, and glucose intolerance after intracerebroventricular injection, and FGF receptors 1 and 4 are present in the hypothalamus of rats and upregulated by high-fat diet [6]. FGFR4 is also present in the medial habenular nucleus [7] adjacent to the pineal gland, suggesting a potential role of FGF15/19 signaling in pain processing, reproductive behavior, nutrition, sleep-wake cycles, stress responses, and learning. Thus, FGF15/19 from the periphery might have crossed the blood–brain barrier (BBB) to exert metabolic effects in adult animals.

It has been long debated whether ingestive and metabolic peptides exert prominent CNS effects by acting through circumventricular organs (CVOs). In contrast to the vast exchange interface of the BBB, the CVOs serve unique regulatory functions. However, there is an astrocytic barrier between the area postrema (a CVO) and nucleus tractus solitarius (inside the BBB) [8], and a tanycyte barrier between the medium eminence (a CVO) and the arcuate nucleus of the hypothalamus [9,10]. There are several ways by which peptides and small proteins interact with the BBB. The mechanisms of BBB permeation, either by specific saturable transport systems or passive diffusion, would affect strategies for drug delivery as well as physiological consequences of neuroendocrine crosstalk [11,12]. Among the FGF family of growth factors, basic FGF (FGF2) is transported across the BBB by adsorptive transcytosis [13], whereas FGF21 crosses the BBB by simple diffusion without saturable influx [14]. Most recently it was shown that circulating FGF19 is associated with remission of diabetes after Roux-en-Y gastric bypass surgery for obesity [15]. With the renewed interest in FGF15/19 in feeding and metabolic activities, we quantified its ability to enter the brain from the periphery.

## Materials and methods

### Animals and chemicals

Three-month-old male CD1 mice were purchased from Charles River Laboratories International (Wilmington, MA, USA,) and maintained at the animal care facility with food and water *ad libitum* in a 12 h light/12 h dark lighting schedule. The mice were used following a protocol approved by the Institutional Animal Care and Use Committee.

Human recombinant FGF19 and mouse recombinant FGF15 were purchased from Prospec (Ness Ziona, Israel). Bovine serum albumin (BSA) and chloramine-T reagents for radioactive labeling were obtained from Sigma (St. Louis, MO, USA). Iodination of FGF19 with ^125^I (PerkinElmer, Shelton, CT, USA) and BSA with ^131^I (PerkinElmer) was achieved by the chloramine-T method. The radioactively iodinated peptides were purified on a column of Sephadex G-10 (Sigma). Acid precipitation indicated that 99.4% of the ^125^I in the purified labeled peptides was incorporated to FGF19 (^125^I-FGF19), while the ratio for ^131^I and BSA (^131^I-albumin) was 99.7%. The specific activities of ^125^I-FGF19 and ^131^I-albumin were 1.23 × 10^8^ cpm/μg (2.05 MBq/μg) and 1.18 × 10^8^ cpm/μg (1.97 MBq/μg), respectively.

### Test of stability of FGF19 in mouse

#### Reversed phase high performance liquid chromatography (HPLC)

The stability of FGF19 in the brain and circulation at 10 min was determined in brain homogenate and serum samples (n = 3). After anesthesia, each mouse received an iv bolus injection of 100 μl lactated Ringer’s solution containing radioactively labeled tracers (6.8 × 10^6^ cpm, or 0.113 MBq of ^125^I-FGF19 and 3.6 × 10^6^ cpm, or 0.06 MBq of ^131^I-albumin) and 1% BSA. The mouse was decapitated 10 min after injection. Blood was collected from the right carotid artery into an ice-chilled tube immediately before decapitation. The brain was quickly removed and homogenized on ice in 1 ml cold phosphate-buffered saline (PBS) in the presence of 2× Halt™ protease inhibitor cocktail (Pierce, Rockford, IL, USA). For control experiments, brain from individual naïve mice was homogenized in PBS containing about 3,500 cpm (5.8 × 10^-5^ MBq) of ^125^I-FGF19 and 6,000 cpm (0.0001 MBq) of ^131^I-albumin. The amount of radioactivity was similar to that in the brain 10 min after iv injection. The homogenate was centrifuged at 18,000 g at 4°C for 20 min. Supernatant from two brains was combined and filtered through a 0.22 μm syringe filter (Fisher Scientific, Pittsburgh, PA) for HPLC analysis. Blood was kept at 4°C overnight before centrifugation at 1,000 g at 4°C for 20 min to obtain serum. Supernatant (about 5,100 cpm, 8.5 × 10^-5^ MBq) and 18 μl of serum (about 7,800 cpm, 0.00013 MBq) were diluted separately with adequate amounts of HPLC-grade water (Sigma) containing 0.1% trifluoroacetic acid (Applied Biosystems, Grand Island, NY, USA) so that the final volume of each sample was about 1 ml for loading onto the 1-ml sample loop of the HPLC. Separation was achieved with a protein C4 column (Vydac, Hesperia, CA, USA). The mobile phase (containing 0.1% trifluoroacetic acid) increased from 10% acetonitrile (EMD Chemicals Inc, Gibbstown, NJ, USA) linearly over 40 min with a flow rate of 1 ml/min. Forty fractions were collected at a rate of 1 fraction/min. The radioactivity of each fraction was measured with a dual-channel program in a Wallac 1470 Wizzard γ-counter. The result was plotted as the radioactivity of each fraction vs time. The fractions with the maximum cpm value of the peaks on the graph were further examined by acid precipitation for confirmation.

#### Acid precipitation

The brains and sera collected at 5, 10, and 20 min after iv injection of ^125^I-FGF19 and ^131^I-albumin for the multiple-time regression analysis (shown below) were used for acid precipitation analysis. The brains were processed as mentioned above to obtain the supernatant. Serum was diluted with adequate amounts of PBS containing 1% BSA before acid precipitation. Equal volumes of 30% trichloroacetic acid (3 parts 50% trichloroacetic acid in water + 2 parts brine) was added to the sample and vortexed. After incubation on ice, the mixture was centrifuged at 3,400 g at 4°C for 20 min. The supernatant and precipitate were separated and the radioactivity measured with a dual-channel program γ-counter. Acid precipitability was defined as the percent of radioactivity in the total precipitate.

### Kinetics of FGF19 influx from blood to brain

The kinetics of FGF19 transferring from blood to brain across the BBB was determined by multiple-time regression analysis and in-situ brain perfusion [16] with modifications as described previously [17,18]. The rate and amount of blood-to-brain transfer of ^125^I-FGF19 in the presence or absence of excess unlabeled FGF19 or FGF15 were compared with those of the vascular marker ^131^I-albumin.

#### Multiple-time regression analysis

Two groups of mice were studied simultaneously. One group of mice (n = 8) received iv injection of 100 μl lactated Ringer’s solution containing 4.3 × 10^6^ cpm (0.072 MBq) of ^125^I-FGF19, 2.5 × 10^6^ cpm (0.042 MBq) of ^131^I-albumin and 1% BSA at time 0. At 1, 3, 5, 7, 10, 13, 16 or 20 min (the experimental time) after injection, one mouse per time point was decapitated to collect brain. Blood was also collected from the right carotid artery immediately before decapitation to obtain serum. To determine whether FGF19 transport across BBB is a saturable process, a second group of mice (n = 8) was treated the same as the first group except that the injection solution contained an additional 2 μg/mouse unlabeled FGF19 (about 56-fold excess to ^125^I-FGF19). Radioactivity in brain and 50 μl serum was measured with a dual-channel program on the γ-counter. For comparison and measurement of peripheral tissue uptake, liver and kidney were also sampled.

Based on the exponentially decreasing pattern of ^125^I radioactivity in serum over experimental time resulting from the decline of ^125^I-FGF19 in blood, the exposure time τ was calculated as the integral of serum radioactivity from time 0 to experimental time t divided by the serum radioactivity at experimental time t [18]. It represents the theoretical time needed for the tissue to accumulate ^125^I radioactivity from blood to the level measured at experimental time t under a hypothetical condition in which serum radioactivity remains constant at the same level as at experimental time t throughout the experiment. The radioactivity in the tissue (cpm) was normalized to its weight (g) and to the radioactivity in the serum (cpm/μl), and thus expressed as the tissue/serum ratio (μl/g). The linear regression correlation between the tissue/serum ratio and the exposure time (for ^125^I-FGF19) or the experimental time (for ^131^I-albumin) was determined with GraphPad Prism software (San Diego, CA). The unidirectional influx rate (Ki, μl/g-min) and the initial volume of distribution (Vi, μl/g) were defined as the slope and the Y-axis intercept from the regression line, respectively.

#### In-situ brain perfusion

To study the kinetics of FGF19 transport across BBB without interference from peripheral factors in the circulation, 3 groups of mice (n = 10 /group) were transcardially perfused with oxygenated modified Krebs buffer containing ^125^I-FGF19 and ^131^I-albumin (1000 cpm/μl, 1.67 × 10^-5^ MBq) for 0.5, 1, 1.5, 2, 2.5, 3, 3.5, 4, 4.5 or 5 min (one mouse per time point). The perfusion of radioisotopes was preceded by a 2-min pre-wash and followed by a 1-min post-wash with the same oxygenated modified Krebs buffer without radioactively labeled tracers. The flow rate was 2 ml/min, and perfusion was driven by a syringe perfusion pump. The descending aorta was completely clamped by a hemostat throughout the perfusion. Mice were decapitated immediately at the end of the post-wash. The “hot only” control group received only radioactively labeled tracers. To determine whether FGF19 transport from blood to brain is saturable, two additional groups of mice (n = 10/group) were perfused in the presence of 50-fold excess unlabeled FGF19 (0.38 μg/ml) and FGF15 (0.38 μg/ml), respectively, in perfusion buffer with radioactively labeled tracers. The recipe of the modified Krebs buffer is (g/L): NaCl 7.19, KCl 0.3, CaCl_2_ 0.28, NaHCO_3_ 2.1, KH_2_PO_4_ 0.16, MgCl_2_▪6H_2_O 0.37, D-glucose 0.99 (pH = 7.4) with 1% BSA. The brain was collected for measurement of radioactivity in the γ-counter. The radioactivity of the tissue (cpm) was normalized to its weight (g) and the radioactivity of the radioactively labeled tracers (cpm/μl); the result was expressed as tissue/perfusate ratio (μl/g). The brain/perfusate ratio of ^125^I-FGF19 was further normalized to that of ^131^I-albumin by subtraction. The linear regression correlation analysis between normalized brain/perfusate ratio and experimental time was the same as described for the multiple time regression analysis.

### Statistics

The linear regression line of each group in the kinetic assays, whether Ki is significantly different from zero (a condition of no influx), and the differences between the regression lines in Ki and Vi were compared by the least square method with GraphPad Prism software (La Jolla, CA, USA).

## Results

### FGF19 is stable in both blood and brain

**Figure 1:**
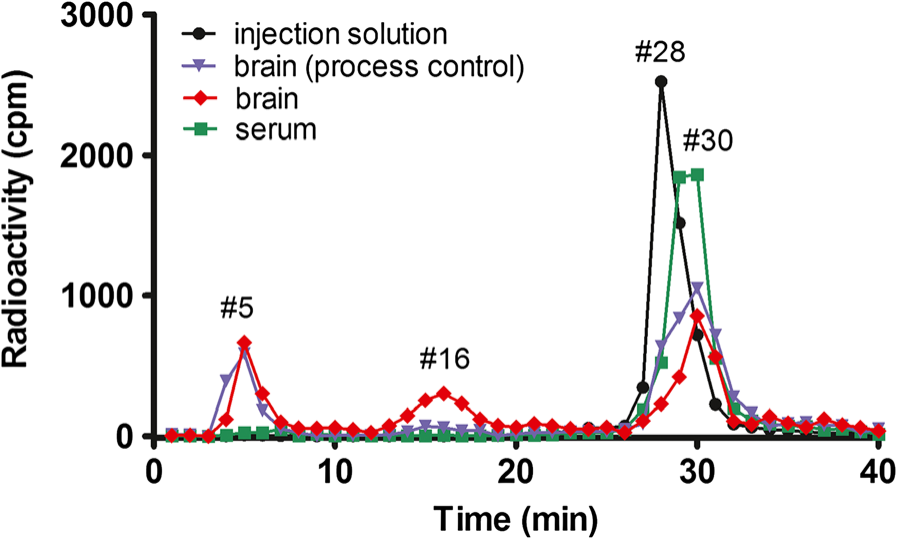
**HPLC chromatogram of the degradation profiles of **^**125**^**I-FGF19 in brain and serum 10 min after iv injection.** The first fraction at the retention time of 5 min represents free ^125^I, the second fraction at retention time of 16 min represents small fragments of degraded ^125^I-FGF19, and the last fraction at retention time 28–30 shows intact ^125^I-FGF19. Most radioactivity in the serum coincided with that of the injection solution. The elution profile of brain supernatant in mice 10 min after iv injection of ^125^I-FGF19 was similar to that of *ex-vivo* processing (process control) where ^125^I-FGF19 was added in the homogenizer. The results indicate that ^125^I-FGF19 remained intact in the blood and brain compartments at this time (n = 3 /group).

The degradation patterns of ^125^I-FGF19 in brain and blood 10 min after iv delivery were determined by HPLC (Figure 1). The retention time of intact ^125^I-FGF19 was 28 min, shown as a single peak in the chromatogram of the injection solution. Acid precipitation of fraction #28 (96.75%) also confirmed that the peak represented intact ^125^I-FGF19. The ^125^I-FGF19 chromatogram of serum was similar to that of the injection solution with only one major peak, indicating that FGF19 was relatively stable in serum for 10 min. However, there were three peaks in the chromatogram of brain: at 5, 16, and 30 min. Further acid precipitation assay of these three fractions (#5, #16, #30) showed that the precipitation rates were 0.72%, 1.58%, and 92.59%, respectively. The chromatogram of the brain processing control was similar, suggesting that most of the degradation occurred *ex-vivo* during tissue processing. Analysis of the area under the peak indicated that 10 min after iv delivery, 99.76% of the ^125^I-FGF19 remained intact in the serum. Free ^125^I accounted for 0.04% of total radioactivity eluted whereas small degradation fragments accounted for only 0.02%. In brain, 93.37% of the ^125^I-FGF19 remained intact, even without normalization with the processing control. Of the total radioactivity, 2.55% represented free ^125^I, where 3.35% were degraded fragments. ^131^I-albumin was relatively stable in both brain and blood (data not shown).

**Figure 2:**
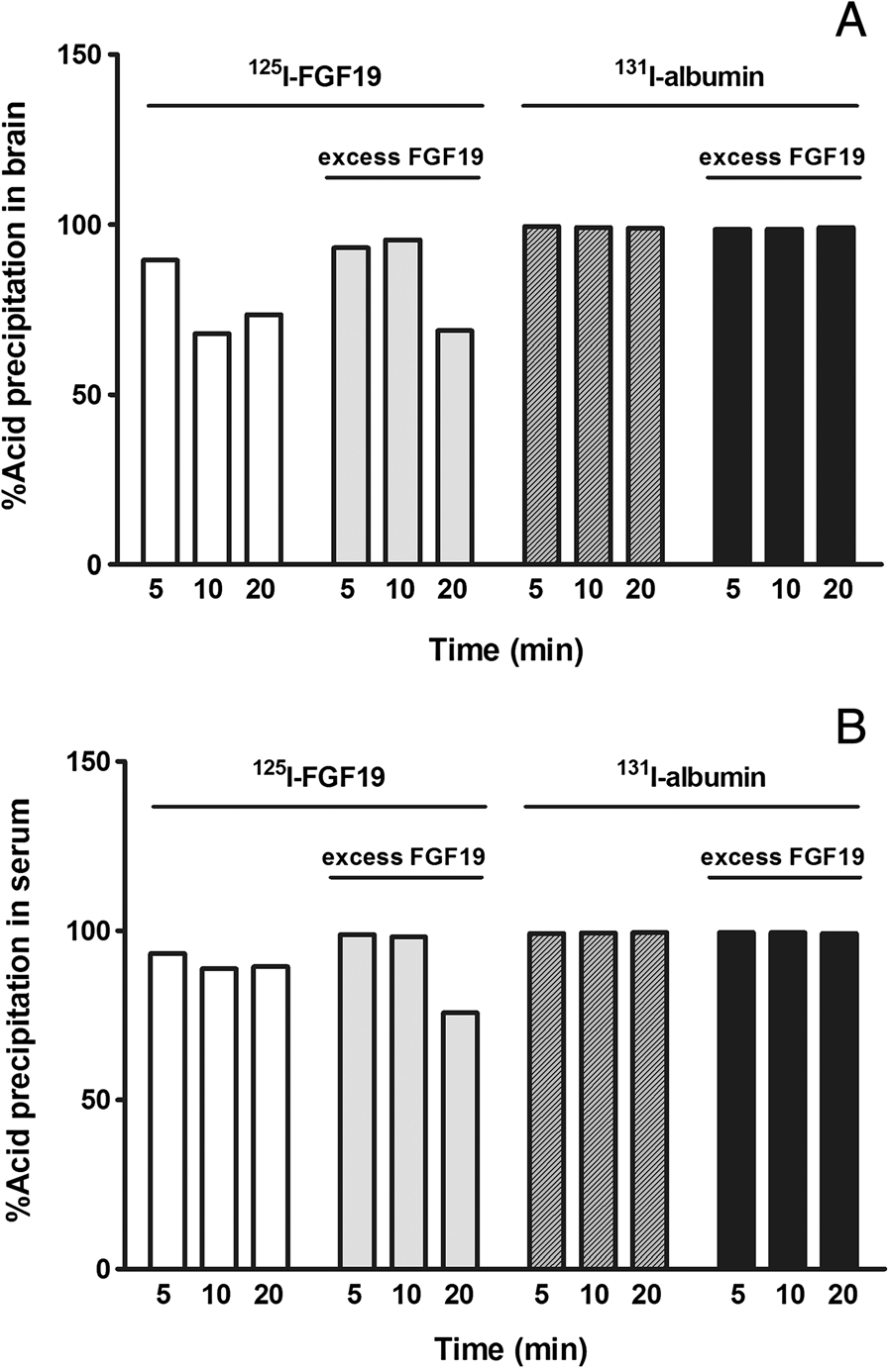
**Acid precipitation rate of **^**125**^**I-FGF19 and **^**131**^**I-albumin at 5, 10, and 20 min after iv delivery with or without the presence of 56-fold excess unlabeled FGF19 (one mouse for each time point). (A)** In the brain, the acid precipitability of ^125^I-FGF19 was decreased at 10 and 20 min in comparison with the 5 min sample. The presence of excess unlabeled FGF19 helped to maintain the high acid precipitability of ^125^I-FGF19 at 10 min but not at 20 min. The acid precipitability of ^131^I-albumin remained high throughout the study. **(B)** In serum, the decline of acid precipitability of ^125^I-FGF19 was mainly seen at 20 min in the group receiving excess unlabeled FGF19.

Acid precipitation analysis is not as sensitive as HPLC, but it offers a simple and fast way to check the integrity of radioactively labeled small proteins. In brain, the acid precipitability of ^125^I-FGF19 decreased from 89.52% at 5 min to 67.89% at 10 min after iv injection, and remained about 70% up to 20 min. The lower precipitability at 10 min might be aberrant and it was lower than that seen by HPLC. In the presence of 56-fold excess unlabeled FGF19 (the amount used was limited by cost), the precipitability remained above 93% at 10 min, and decreased to 70% at 20 min after iv delivery (Figure 2A). In contrast with the ^125^I-FGF19 in the brain, that in the serum was more stable as shown by acid precipitability above 89% at 20 min. With excess unlabeled FGF19, the precipitability of ^125^I-FGF19 in serum remained more than 98% at 10 min, and decreased to 76% at 20 min. The precipitability of ^131^I-albmin either in brain or in serum was more than 99% up to 20 min (Figure 2B). Both HPLC and acid precipitation data indicate that FGF19 detected in serum and in brain homogenate was stable, with detectable degradation in the brain homogenate at 10 min, and that the excess FGF19 slowed the degradation process.

### Excess FGF19 extends the half-life of ^125^I-FGF19 in blood

**Figure 3:**
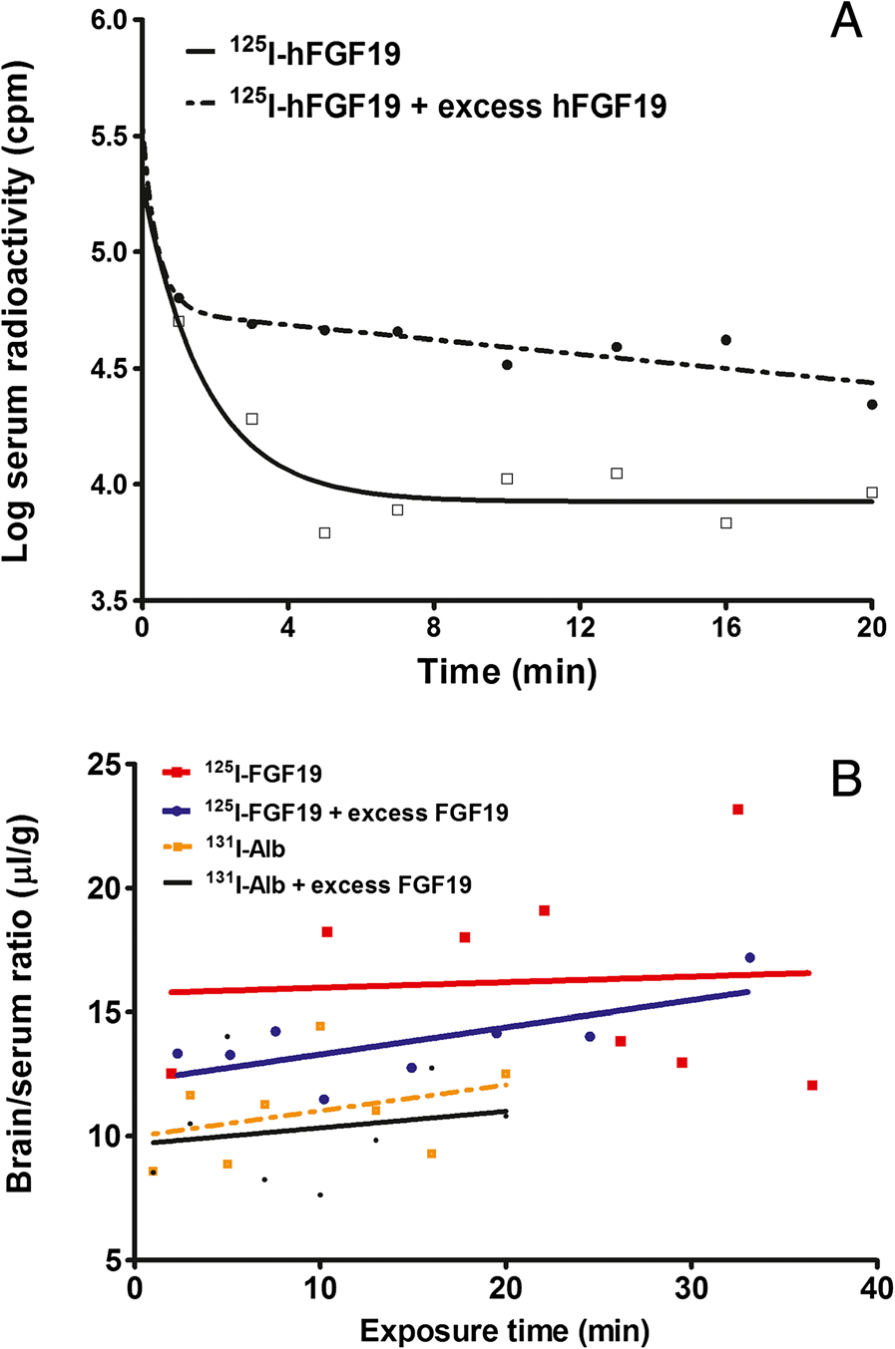
**Multiple time-regression analysis of **^**125**^**I-FGF19 transport from blood to brain. (A)** The ^125^I-FGF19 clearance profile in blood was fitted with a two-phase exponential disappearance model. In the presence of 56-fold excess unlabeled FGF19, the area under curve of ^125^I-FGF19 was increased and the half-life of the slow phase of clearance was prolonged. **(B)** The influx rate Ki of ^125^I-FGF19 from blood to brain was not significant although the initial volume of distribution Vi was significantly higher than that of the co-administered vascular space marker ^131^I-albumin. The presence of excess unlabeled FGF19 resulted in the influx of ^125^I-FGF19 (p = 0.05). The influx of ^131^I-albumin remained non-significant. Each time point indicates an individual mouse (n = 8 /group).

As shown in Figure 3A, a two-phase exponential disappearance model best fit the pattern of ^125^I-FGF19 in the blood over a time course of 20 min. The half-time disappearance of ^125^I-FGF19 was 0.90 min in the fast phase and 9.2 min in the slow phase. Co-injection of 56-fold excess unlabeled FGF19 decreased the fast phase half-life of ^125^I-FGF19 to 0.34 min, but increased the slow phase half-life to 143.5 min.

### The influx of ^125^I-FGF19 from blood to brain is limited and unmodified by excess FGF19

Determined by multiple-time regression analysis, the unidirectional influx rate of ^125^I-FGF19 from blood to brain was not significantly different from zero (Ki = 0.02 ± 0.14 μl/g-min; F_1,6_ = 0.03, p = 0.88). With co-administration of 56-fold excess FGF19, the influx of ^125^I-FGF19 was borderline significant (Ki = 0.11 ± 0.05 μl/g-min; F_1,6_ = 5.72, p = 0.05). Although comparison of the influx rate of ^125^I-FGF19 with and without the presence of excess FGF19 was not significant (F_1,12_ = 0.32, p = 0.58), the linear correlation was stronger (r = 0.7, in contrast to the “hot only” group r = 0.07). This coincided with a greater area under curve and longer half-life of the slow phase of ^125^I-FGF19 disappearance in blood. Thus, in the presence of excess unlabeled FGF19, more ^125^I-FGF19 was available to reach the brain compartment. The initial volume of distribution of ^125^I-FGF19 (Vi = 15.75 ± 3.44 μl/g), though not decreased significantly (F_1,13_ = 1.46, p = 0.25) by unlabeled FGF19 (Vi = 12.21 ± 0.21 μl/g), was significantly higher than that of albumin (Vi = 9.98 ± 1.30 μl/g) (F_1,13_ = 5.6, p < 0.05). Nonetheless, the influx rate of the vascular marker ^131^I-albumin was not significantly different from either 0 or ^125^I-FGF19 (Figure 3B).

**Figure 4:**
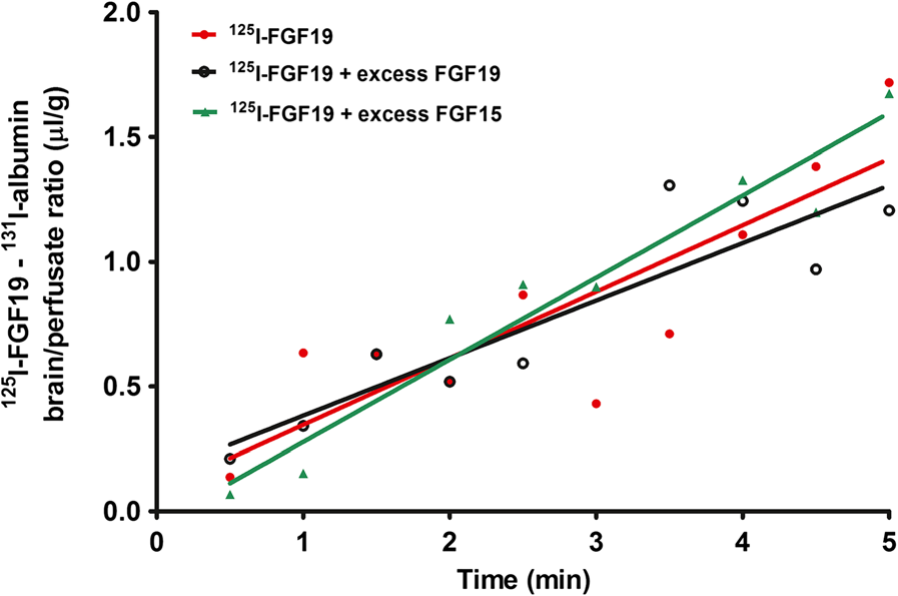
**In the in-situ brain perfusion study, there was significant influx of **^**125**^**I-FGF19 (values already normalized by subtraction of **^**131**^**I-albumin in the same mouse).** Each data point represents one mouse. The presence of 50-fold excess FGF19 or FGF15 did not change the influx of ^125^I-FGF19.

The results from multiple-time regression analysis after iv bolus injection of FGF19 may be affected by serum binding proteins. Therefore, we further performed in-situ brain perfusion with blood-free physiological buffer. There was a significant influx of ^125^I-FGF19 from perfusate to brain (Ki = 0.27 ± 0.06 μl/g-min, F_1,8_ = 22.06, p = 0.002). However, the presence of 50-fold excess of either FGF19 or FGF15 did not change the influx rate of ^125^I-FGF19. The Ki for ^125^I-FGF19 was 0.23 ± 0.04 μl/g-min in the presence of excess unlabeled FGF19, and 0.33 ± 0.03 μl/g-min in the presence of excess unlabeled FGF15. The slopes of these three regression lines were not significantly different from each other (F_2,21_ = 1.10, p = 0.35). Similarly, the excess of FGF19 or FGF15 did not change the volume of distribution of ^125^I-FGF19 (F_2,23_ = 0.24, p = 0.79) (Figure 4), the values already being normalized by subtraction of albumin space at each time point. The results suggest that FGF19 had a low level of permeation across the BBB in a process that is probably non-saturable, as the amount of excess FGF19 or FGF15 used in this study was not able to modulate the influx.

### Influx of ^125^I-FGF19 from blood to peripheral organs is also affected by excess FGF19

**Figure 5:**
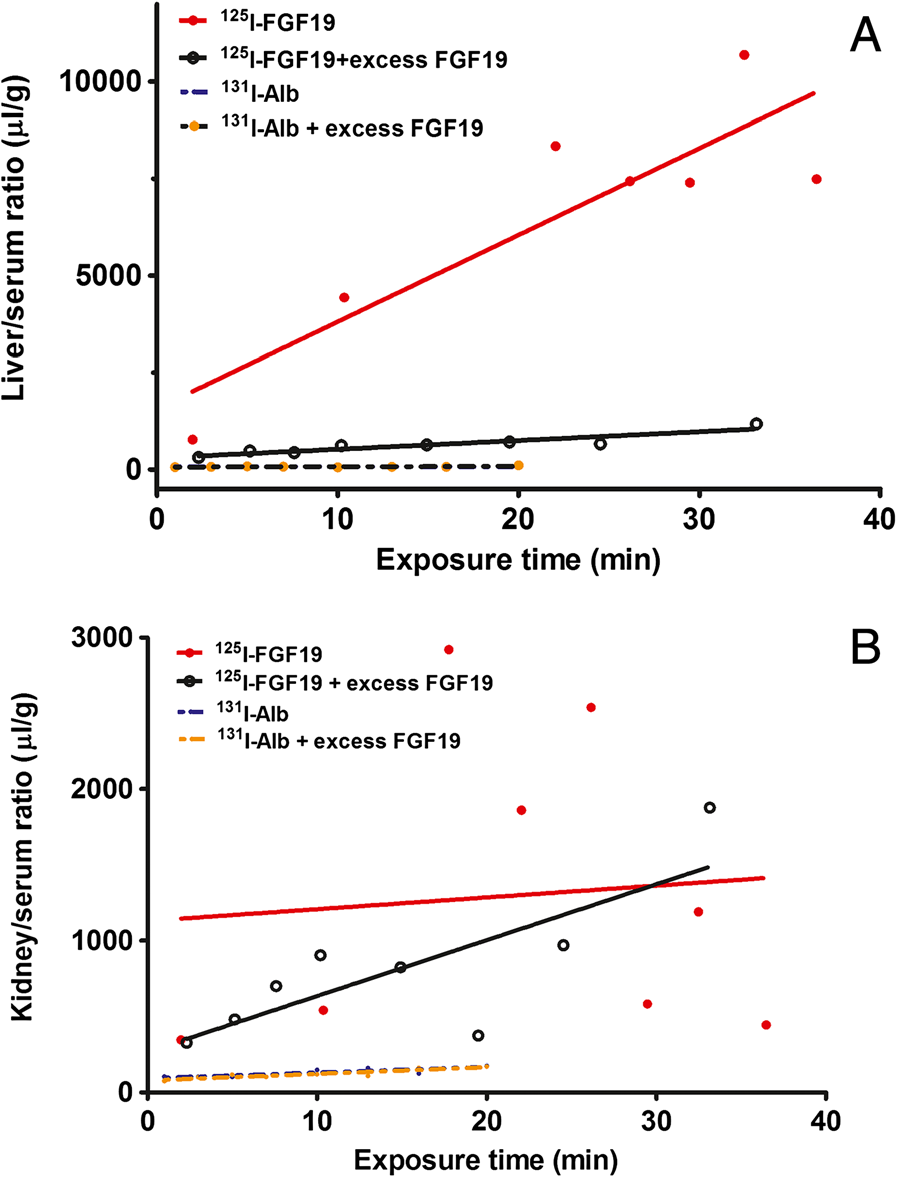
**The influx of **^**125**^**I-FGF19 from blood to peripheral organs after iv injection. (A)** In the liver, there was a rapid influx of ^125^I-FGF19 (Ki = 223.60 ± 55.47 μl/g-min). The presence of 56-fold excess unlabeled FGF19 produced a significant reduction of the Ki which remained linear and higher than that of the vascular space marker ^131^I-albumin. **(B)** In the kidney, the basal uptake of ^125^I-FGF19 was highly variable and not significantly different from zero. The presence of excess unlabeled FGF19 resulted in a linear clearance of ^125^I-FGF19 but did not change the minimal uptake of ^131^I-albumin. Each time point indicates an individual mouse (n = 8/group).

^125^I-FGF19 had an influx rate of 223.60 ± 55.47 μl/g-min from blood to liver, significantly higher than zero (F_1,5_ = 16.26, p = 0.01). In the presence of excess unlabeled FGF19, the Ki was reduced to 22.61 ± 3.82 μl/g-min, although the influx remained significant compared with zero (F_1,6_ = 34.96, p = 0.001). The difference in the liver uptake of ^125^I-FGF19 between the two groups was significant (F_1,11_ = 13.11, p = 0.004) (Figure 5A).

The influx of ^125^I-FGF19 to kidney showed large variation among data points, and there was no significant linear regression (Ki = 7.78 ± 35.52 μl/g-min. F_1,6_ = 0.05, p = 0.83). Contrary to the liver where co-injection of excess FGF19 inhibited the hepatic uptake of ^125^I-FGF19, the kidney showed a greater influx of ^125^I-FGF19 in the presence of 56-fold excess of unlabeled FGF19. The Ki was 36.92 ± 11.89 μl/g-min (F_1,6_ = 9.65, p = 0.02) (Figure 5B). The results suggest that FGF19 processing by the liver and kidney is dependent on the amount of FGF19 available in blood.

## Discussion

We first showed that human FGF19 was stable in mouse blood and its half-time disappearance was further extended by excess FGF19. In the “hot only” group, there was no meaningful permeation of ^125^I-FGF19 into brain as seen by multiple-time regression analysis. The initial volume of distribution of ^125^I-FGF19 remained higher than that of co-administered ^131^I-albumin. The presence of FGF19 in the cerebral vasculature may allow its binding and activation of BBB endothelia and thus induce CNS function indirectly without crossing the BBB, as seen with adiponectin [19]. Nevertheless, the small portion of FGF19 entering the brain was stable for at least 10 min, so that it could exert direct CNS effects.

The influx of ^125^I-FGF19 became significant in the presence of excess unlabeled FGF19 or its homolog FGF15 both by multiple-time regression analysis after iv injection and by *in-situ* brain perfusion. Because of the low level of permeation without an increase of Vi over time, we did not perform capillary depletion to further determine the compartmental distribution of FGF19 loosely adherent to microvessels, entrapped in endothelia, or already transcytosed across the BBB to reach brain parenchyma. However, based on the linearity of brain uptake over time, we expect that a significant amount of ^125^I-FGF19 reached brain parenchyma in the group receiving excess unlabeled FGF19. Differences between the iv and *in-situ* brain perfusion studies were probably caused by blood-borne factors and differential tissue distribution. Indeed, the serum half-life of ^125^I-FGF19 was increased by the presence of excess unlabeled FGF19, particularly during the slow phase of distribution. This coincided with a larger area under curve of ^125^I-FGF19, suggesting that more ^125^I-FGF19 was available to reach the brain compartment. This probably occurred at least partially from the excess unlabeled FGF19 saturating peripheral uptake or degradation processes.

To determine a possible effect of peripheral tissue distribution of FGF19 entry into brain, we determined its uptake by liver and kidney. There was a high influx rate of FGF19 from blood to liver, consistent with the liver being a major target organ for FGF15/19 [4,20,21]. In the presence of excess unlabeled FGF19, the influx rate of FGF19 to the liver was significantly attenuated. This suggests the presence of a specific, saturable process. Uptake of FGF19 by the liver may be mediated by both conduit capillaries (reflected by albumin space) and exchange capillaries; the latter probably represent the fraction attenuated by excess unlabeled FGF19. Receptors for FGF15/19 include FGF receptors (particularly R4) and α- and β-Klotho proteins [22,23]. The expression of Klotho proteins are tightly regulated [24]. They are the most probable mediators of regulated liver uptake of ^125^I-FGF19 in this study.

β-Klotho plays an important role in regulating transient receptor potential vanilloid 5 (TRPV5) in the kidneys [25]. The kidney is clearly involved in the clearance of FGF19, as blood concentrations of FGF19 are higher in subjects with end stage renal disease [26]. However, the influx rate of FGF19 to the kidney of normal healthy mice was non-linear during the study period of 1–20 min. Although there have not been comparative studies, differences in the level of expression of these FGF15/19 binding proteins in the liver and kidney might underlie the difference in the organ-specific influx rate. The saturation of FGF receptors and Klotho in the liver by excess unlabeled FGF19 could have reduced the influx of ^125^I-FGF19, whereas the simultaneous displacement and increased blood concentration of ^125^I-FGF19 may enable a linear renal excretion.

The low permeability of FGF19 across the BBB contrasts with that of FGF21, which shows significant influx by simple diffusion across the BBB [14]. FGF15/19 and FGF21 belong to the class of endocrine FGF small proteins, as their weak interaction with heparin sulfate enables them to diffuse away from the site of production. However, unlike FGF21 that is mainly secreted from the liver after prolonged fasting to exert glucagon-like effects, FGF15/19 is produced by the small intestine in response to feeding and shows insulin-like actions [21,27]. We do not anticipate a major difference of BBB interactions between the homologous proteins FGF15 and FGF19 that show 53% structural identity [2]; this is supported by the lack of effect of excess unlabeled FGF15 to modulate the influx of ^125^I-FGF19.

Although only a trace amount of ^125^I-FGF19 was used in this study, the concentration remains higher than that of endogenous FGF15 in mice or FGF19 in humans. Thus, peripheral actions of FGF15/19 in metabolic regulation may dominate over CNS effects in the basal state. It is not clear whether peripheral FGF15/19 plays a prominent role to induce CNS effects in metabolic signaling and obesity [6]. However, the initial volume of distribution, Vi, of ^125^I-FGF19 was higher than that of ^131^I-albumin, supporting the possibility that FGF19 modifies BBB endothelial function. There are also developmental changes of FGF19 gene expression in brain microvessels collected from 2-month-old to 8-year-old humans [28]. Though it is not clear whether these transcripts are translated, and though adult brain does not express FGF19 [29], it is apparent that FGF19 can affect CNS function on either side of the BBB.

## Conclusion

In summary, we showed in normal mice that ^125^I-FGF19 remained stable in blood and brain homogenate at least for 10 min. It did not have significant entry into brain after iv injection, but there was increased permeation in the presence of excess unlabeled FGF19 and during *in-situ* brain perfusion. In the presence of excess FGF19, the first rapid phase clearance of ^125^I-FGF19 in serum was shortened and the second slow phase was remarkably prolonged. ^125^I-FGF19 had a fast uptake by the liver, and this was significantly attenuated by excess FGF19. In the presence of excess FGF19, the low level of kidney excretion of ^125^I-FGF19 showed a linear acceleration. Thus, the pharmacokinetics of FGF19 entry into brain is influenced by its serum concentration, and shows significant hepatic and renal interactions.

## Competing interests

The authors declared that they have no competing interest.

## Authors’ contributions

HH performed the study and wrote part of the paper. WP performed part of the study and completed the first and last draft of the paper. AJK conceived the study and wrote the paper. All authors have read and approved the final version of the manuscript.
